# ﻿A new species of *Sedum* (Crassulaceae, Saxifragineae) from Guangxi, China

**DOI:** 10.3897/phytokeys.252.128011

**Published:** 2025-02-20

**Authors:** Chun-Yu Zou, Tao Meng, Shi-Yong Meng, Yan Liu

**Affiliations:** 1 Guangxi Key Laboratory of Plant Conservation and Restoration Ecology in Karst Terrain, Guangxi Institute of Botany, Guangxi Zhuang Autonomous Region and Chinese Academy of Sciences, Guilin, 541006, Guangxi, China Guangxi Zhuang Autonomous Region and Chinese Academy of Sciences Guilin China; 2 Laboratory of Systematic Evolution and Biogeography of Woody Plants, College of Nature Conservation, Beijing Forestry University, Beijing, 100871, China Beijing Forestry University Beijing China; 3 College of Life Sciences, Peking University, Beijing, 100871, China Peking University Beijing China

**Keywords:** Phylogeny, sect. *Sedum*, *Sedumguangxiense*, taxonomy

## Abstract

A new species, *Sedumguangxiense***sp. nov.**, discovered in Guangxi Province, China, is described and illustrated. Molecular phylogenetic analyses and morphological differences indicate that this species is well separated from its close relatives in *Sedum*, justifying its recognition as a distinct new species. Morphologically, it closely resembles *Sedumtosaense* and *S.emarginatum* in its leaf shape, inflorescence type and life form, but it can be easily distinguished in its erect stems when young, much larger leaves, narrowly triangular petals, ovate nectar scales and oblong anthers.

## ﻿Introduction

The genus *Sedum*Linnaeus (1753: 430) (stonecrops) comprises ca. 470 species mainly distributed in the Northern Hemisphere, but some extend to the Southern Hemisphere in Africa and South America (Fu and Ohba 2001). It typically thrives in open, climatically (semi-)arid environments and comprises herbs to (sub-)shrubs with succulent leaves (Stephenson 1994). The molecular study by Messerschmid et al. (2020) showed that *Sedum* is highly polyphyletic and, in order to render *Sedum* monophyletic, about 14 genera derived from within *Sedum* would have to be merged with it. This broader concept of *Sedum* would encompass approximately 755 species, mainly distributed in the Mediterranean, Central America, the Himalayas and East Asia (Stephenson 1994; Thiede and Eggli 2007).

In China, there are approximately 120 species of *Sedum* including 91 endemics which are particularly abundant in the south-western region. Sedum is classified into sect. Filipes (Fröd.) S.H. Fu (1965: 115), sect. Oreades (Fröd.) K.T. Fu (1974: 52) and sect. Sedum (Fu and Ohba 2001) which is by far the largest. China stands out as one of the primary centres of biodiversity for *Sedum*, with several studies undertaken (Fu 1965, 1974; Rao 1996; Fu and Ohba 2001). However, ongoing exploration reveals new species within the *Sedum* genus in China, indicating that the full extent of its diversity is yet to be uncovered (Zou et al. 2020; Ying 2022a, 2022b; Huang et al. 2023; Meng et al. 2023).

Guangxi Province, located in southern China, boasts a significant biodiversity. The challenge of accurately identifying *Sedum* species within this region is difficult due to the presence of over 17 species. Notably, during the taxonomic revision of *Sedum* in Guangxi, anomalous specimens were collected in Yongfu County, Guangxi Province, China, in 2013. Subsequently, a field survey was conducted in May 2020 to investigate these unidentified specimens. Field observations suggested similarities to *S.tosaense*Makino (1892: 52). However, a thorough examination of both living and herbarium specimens revealed distinctions from *S.tosaense*, particularly in the erect stems during early growth stages, petal morphology, nectar scale shape and style length.

During a field survey of plant resources in Fangcheng City, Guangxi, China, in April 2021, populations of an unknown *Sedum* species were discovered in forests. Through field observations and detailed comparisons, we concluded that this plant is conspecific with the one discovered in Yongfu County, establishing it as a putatively undescribed species. This study intends to determine the phylogenetic position of these plants and to provide a comparative morphological analyses with its close relatives within the genus *Sedum*. To achieve this, phylogenetic analyses were conducted using molecular sequence data from nuclear markers (nrITS) within Eastern Asian Sedumsect.Sedum. In this study, we clarify the status of the putatively new species by determining its phylogenetic position and comparing its morphology with that of its close relatives.

## ﻿Material and methods

To examine its phylogenetic relationships and confirm the morphological distinctiveness of this new species from other taxa, voucher specimens and living individuals of *S.guangxiense* were collected from Yongfu County and Fangcheng City. We consulted the relevant literature (Fu 1965, 1974; Rao 1996; Fu and Ohba 2001; Wu et al. 2012; Yang et al. 2012; Xie et al. 2014; Ito et al. 2014a, 2014b, 2017a, 2017b, 2020b; Lu et al. 2019; Zou et al. 2020; Ying 2022a, 2022b; Huang et al. 2023; Meng et al. 2023) and studied herbarium specimens at the Herbaria IBK, IBSC, KUN and PE, therewith comparing our new species with all described Eastern Asian species of Sedumsect.Sedum.

To determine the phylogenetic position of the new species, nuclear internal transcribed spacer (ITS) sequences of 62 accessions were utilised representing 61 species (Mort et al. 2002; Mayuzumi and Ohba 2004; Ito et al. 2014a, 2017a, 2017b, 2018, 2020a; Xie et al. 2014; Zou et al. 2020; Kim et al. 2023; Huang et al. 2023), including the putatively new species. Fifty-six species belong to Sedumsect.Sedum, while two species belong to S.sect.Oreades. These were obtained from GenBank and included in the ITS analysis (Table 1). Based on a phylogenetic study of Crassulaceae (Mayuzumi and Ohba 2004), *Aeoniumcastello-paivae*Bolle (1859: 240), *A.gomerense*Praeger (1929: 473), *A.lancerottense*Praeger (1932: 190), *A.viscatum*Bolle (1859: 241), *Greenoviaaizoon*Bolle (1859: 242) and *Phedimustakesimensis* (Nakai) ‘t Hart (1995: 165) were selected as outgroup members (Table 1).

**Table 1.** Taxon, locality, accession number and reference for internal transcribed spacer (ITS) sequences of *Sedum*, *Aeonium*, *Greenovia* and *Phedimus* species registered in the GenBank database. The sequences were used for molecular analyses.

Taxon	Origin	Accession number	References
**Ingroup**			
**SedumsectionOreades**			
*S.trullipetalum*	Nepal	AB088630	Mayuzumi and Ohba (2004)
*S.oreades*	Nepal	AB088632	Mayuzumi and Ohba (2004)
**SedumsectionSedum**			
*S.actinocarpum*	China: Taiwan	LC229265	Ito et al. (2017a)
*S.alfredii*	China: Guangdong	AB930259	Ito et al. (2014a)
*S.arisanense*	Japan	LC229273	Ito et al. (2017b)
*S.baileyi*	China	FJ919935	Unpublished
*S.brachyrinchum*	China: Taiwan	LC229276	Ito et al. (2017b)
*S.boninense*	Japan	LC530821	Ito et al. (2020a)
*S.bulbiferum*	Japan	KM111165	Xie et al. (2014)
*S.danjoense*	Japan	LC260127	Ito et al. (2017a)
*S.emarginatum*	China: Anhui	EU592006	Unpublished
*S.erythrospermum*	China: Taiwan	AB906473	Ito et al. (2014a)
*S.formosanum*	China: Taiwan	AB930271	Ito et al. (2014a)
*S.guangxiense*-FC	China: Guangxi	OL693034	This study
*S.guangxiense*-YF	China: Guangxi	OL693035	This study
*S.hakonense*	Japan	AB930278	Ito et al. (2014a)
*S.hangzhouense*	China: Zhejian	LC260130	Ito et al. (2017b)
*S.japonicum*	Japan	LC229237	Ito et al. (2017a)
*S.jinglanii*	China: Guangdong	OQ162326	Huang et al. (2023)
*S.jiulungshanense*	China: Zhejiang	LC229243	Ito et al. (2017a)
*S.kiangnanense*	China: Zhejiang	LC229244	Ito et al. (2017a)
*S.kwanwuense*	China: Taiwan	LC229293	Ito et al. (2017a)
*S.lineare*	Japan	AB088623	Mayuzumi and Ohba (2004)
*S.lipingense*	China: Guizhou	MN150061	Zhang et al. (2019)
*S.lungtsuanense*	China: Zhejiang	LC260131	Ito et al. (2017b)
*S.mexicanum*	Japan	LC229247	Ito et al. (2017b)
*S.makinoi*	Japan	AB906476	Ito et al. (2014a)
*S.microsepalum*	China: Taiwan	LC229281	Ito et al. (2017a)
*S.morrisonense*	China: Taiwan	AB906477	Ito et al. (2014a)
*S.mukojimense*	Japan	LC530823	Ito et al. (2020a)
*S.multicaule*	Nepal	AB088631	Mayuzumi and Ohba (2004)
*S.nagasakianum*	Japan	LC229249	Ito et al. (2017a)
*S.nanlingense*	China: Guangxi	MN105949	Zou et al. (2020)
*S.nokoense*	China: Taiwan	AB906478	Ito et al. (2014a)
*S.oligospermum*	China	LC229250	Ito et al. (2017a)
*S.onychopetalum*	China: Nanjin	KM111148	Xie et al. (2014)
*S.oryzifolium*	South Korea	KF954525	Unpublished
*S.polytrichoides*	China: Anhui	KM111143	Xie et al. (2014)
*S.rupifragum*	Japan	LC229254	Ito et al. (2017b)
*S.sarmentosum*	Japan	LC229255	Ito et al. (2017a)
*S.satumense*	Japan	LC229256	Ito et al. (2017a)
*S.sekiteiense*	China: Taiwan	LC229295	Ito et al. (2017a)
*S.subtile*	Japan	LC229257	Ito et al. (2017a)
*S.taiwanalpinum*	China: Taiwan	LC229278	Ito et al. (2017a)
*S.taiwanianum*	China: Taiwan	LC229296	Ito et al. (2017a)
*S.tarokoense*	China: Taiwan	LC229298	Ito et al. (2017b)
*S.tetractinum*	China: Zhejiang	LC260135	Ito et al. (2017b)
*S.tianmushanense*	China: Zhejiang	LC229261	Ito et al. (2017a)
*S.tosaense*	Japan	AB088620	Mayuzumi and Ohba (2004)
*S.triactina*	Japan	AB088629	Mayuzumi and Ohba (2004)
*S.tricarpum*	Japan	LC229259	Ito et al. (2017a)
*S.truncatistigmum*	China: Taiwan	LC229304	Ito et al. (2017a)
*S.uniflorum*	Japan	LC530832	Ito et al. (2018)
*S.yabeanum*	Japan	AB906490	Ito et al. (2014a)
*S.zentaro-tashiroi*	Japan	AB088619	Mayuzumi and Ohba (2004)
*S.wenchuanense*	China	ON707681	Unpublished
**Outgroups**			
*Aeoniumcastello-paivae*	Spain: Canary Islands	AY082127	Mort et al. (2002)
*Aeoniumgomerense*	Spain: Canary Islands	AY082133	Mort et al. (2002)
*Aeoniumviscatum*	Spain: Canary Islands	AY082154	Mort et al. (2002)
*Aeoniumlancerottense*	Spain: Canary Islands	AY082143	Mort et al. (2002)
*Greenoviaaizoon*	Spain: Canary Islands	AY082112	Mort et al. (2002)
*Phedimustakesimensis*	Korea	OP346962	Kim et al. (2023)

The total genomic DNA was extracted from silica gel-dried leaf materials using the CTAB protocol (Doyle and Doyle 1987). DNA sequences of nuclear ribosomal ITS were selected as a marker for molecular phylogenetic studies. The nrITS region was amplified using polymerase chain reactions (PCR) with universal primers, following the protocol of Huang et al. (2017). The complete DNA sequences were submitted to GenBank (Table 1). The ITS sequences obtained by PCR were aligned using MUSCLE version 3.8.31 (Edgar 2004) and were adjusted manually in Bioedit 5.0.9 (Hall 1999). A phylogeny was constructed using Maximum Likelihood (ML) and Bayesian Inference (BI). ML analyses were performed using RAxML v.7.0.4 (Stamatakis et al. 2008) and the substitution model was estimated with ModelTest (Posada and Crandall 1998). BI was conducted in MrBayes 3.2.6 (Ronquist et al. 2012) with the optimal substitution model selected by ModelTest (Posada and Crandall 1998) according to the Akaike Information Criterion. All BI analyses were run for 1,000,000 generations, with four chains in two parallel runs and one tree sampled every 5,000 generations. The convergence of two parallel runs was guaranteed by a splitting frequency of less than 0.005. All other parameters were set to default. The first 25% of the sampled trees corresponding to the burn-in period were discarded and the remaining trees were used to construct a majority rule consensus tree.

## ﻿Results

### ﻿Morphological comparison

In morphological terms, two species closely resemble the undescribed species: *S.tosaense* and *S.emarginatum*Migo (1937: 224). It shares similarities with *S.tosaense* in leaf shape, inflorescence type with three branches (each 2-forked) and the basal leaves forming a rosette. However, the newly-discovered species is distinguished by its erect stems when young, narrowly triangular petals, ovate nectar scales, oblong anthers and longer styles. In contrast, the leaves of sterile shoots of *S.tosaense* are rounder, the leaf base is long-attenuate, but shorter, the nectar scales are subquadrangular at the base and the anthers are oblong-ovoid. It is similar to *S.emarginatum* in having spatulate to obovate leaves with a notch or shallow indentation, but differs in having erect stems when young and alternate leaves.

### ﻿Molecular analysis

**Figure 1.**
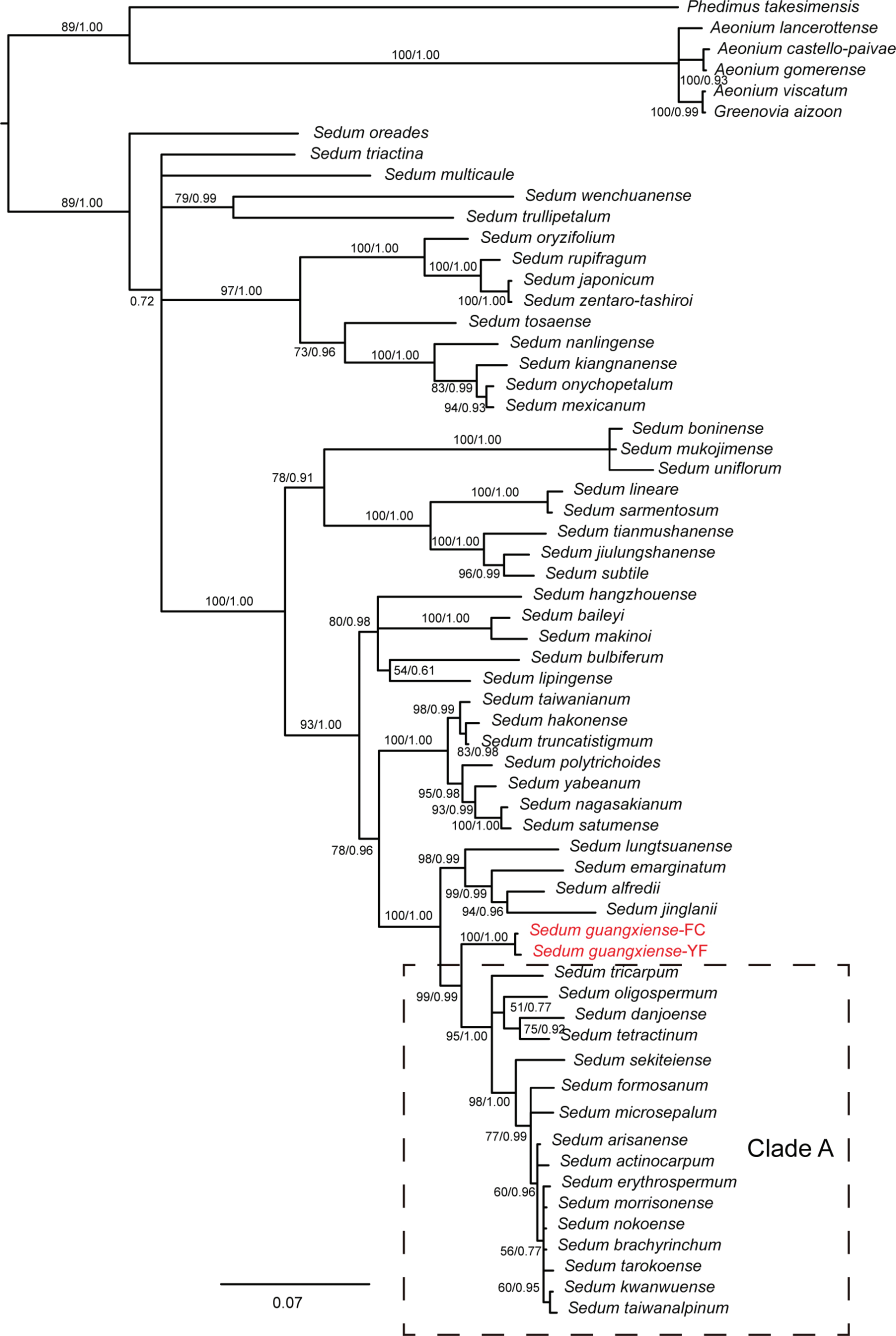
Bayesian phylogenetic tree, based on ITS sequences of Eastern Asian *Sedum*. Bootstrap percentages from the ML analysis (left) and Bayesian posterior probabilities (right) are shown at the nodes.

ML and BI phylogenetic analyses were performed, based on the ITS sequences of 54 *Sedum* species. This set included two ITS sequences from our undescribed species and 53 ITS sequences from GenBank. A 50% majority rule consensus tree of all post-burn-in trees using the optimal substitution model GTR and Bayesian posterior probabilities was generated (PP, Fig. 1). ML analyses were performed with the substitution model GTRGAMMA and 1,000 rapid bootstrap searches. The topology of the ML tree was highly similar to that of the Bayesian tree and the bootstrap support values for this tree were depicted on Fig. 1 (BS). BI and ML analyses confirmed that the putative new species constituted a well resolved clade, sister to a clade with *S.oligospermum*, *S.sekiteiense* and *S.actinocarpum* and 13 further *Sedum* species (PP = 0.99, BS = 99).

## ﻿Discussion

The molecular phylogenetic analysis carried out in this study indicates that *S.guangxiense* constitutes a well-supported distinct clade, which is, with high support, sister to a well-supported clade (named clade A in Fig. 1) which includes *S.oligospermum*, *S.sekiteiense*, *S.actinocarpum* and 13 further species (Fig. 1). However, morphologically, *S.guangxiense* can be readily distinguished by its biennial life form (Table 2), erect stems in youth, larger leaves with a retuse apex, usually 5-merous, but sometimes 4- or 6-merous flowers and longer styles. Although it bears resemblance to *S.tosaense* and *S.emarginatum*, these species are distantly related to *S.guangxiense* in the molecular tree (Fig. 1). Specifically, *S.tosaense* and *S.emarginatum* differ in having a perennial life form, prostrate stems when young, smaller leaves with a rounded and emarginate apex, smaller anthers and shorter styles (Table 2). In addition, *S.guangxiense* (Table 2) differs from *S.tosaense* in its ovate (vs. subquadrangular) nectar scales and from *S.emarginatum* in its alternate leaves (vs. opposite and loosely arranged leaves), strongly unequal (vs. subequal) sepals and ovate (vs. oblong) nectar scales.

**Table 2.** Morphological comparisons between *Sedumguangxiense*, *S.tosaense* and *S.emarginatum*, based on our own measurements of herbarium and living specimens of all three species as well as literature data (Fu and Ohba 2001).

Characters	*S.guangxiense*	*S.tosaense*	*S.emarginatum*
Life form	Biennial	Perennial	Perennial
Flowering stems	Erect when young	Prostrate when young, later erect	Prostrate when young, later erect
Leaf arrangement	Basal leaves forming a rosette	Basal leaves forming a rosette	Basal leaves opposite, loosely arranged
Leaves	Alternate, spatulate to obovate, 1.5–3.5 × 0.5–1 cm, apex usually retuse, rarely round	Alternate, linear-spatulate, 1.2–2 × 0.5–1 cm, apex rounded and emarginate	Opposite, spatulate-obovate to broadly obovate, 1–2 × 0.5–1 cm, apex rounded and emarginate
Inflorescence type	Cyme often 3-branched, each 2-forked	Cyme often 3 (sometimes 4)-branched, each 2-forked	Cymes usually 3-branched, each 2-forked
Flowers	Usually pentamerous, sometimes 4- or 6-merous	Pentamerous	Pentamerous
Carpels	Usually 5, sometimes 4 or 6	5	5
Sepals (colour, shape and size)	Green, spatulate to obovate, strongly unequal, 3–8 × 1–3 mm	Green, linear-spatulate, strongly unequal, 4.5–10 × 2–4.5 mm	Green, spatulate-obovate to broadly obovate, 4–5 × 1–1.5 mm
Petals (colour, shape and size)	Yellow, narrowly triangular, 5–6 × 1.1–1.5 mm	Yellow, narrowly elliptic-lanceolate, 5–6 × 1.5–2.5 mm	Yellow, linear-lanceolate to lanceolate, 6–8 × 1.5–2 mm
Nectar scales (colour, shape and size)	Yellow, ovate, 0.3–0.4 mm	Yellowish, subquadrangular, 0.2–0.4 mm	Yellow, oblong, ca. 0.3–0.6 mm
Anthers (colour, shape and size)	Reddish-brown, oblong, ca. 0.8 mm	Reddish-brown, oblong-ovoid, 0.5–0.6	Reddish-brown, oblong-ovoid, 0.4–0.5
Style length	1.6–2 mm	1–1.2 mm	1.1–1.3 mm
Primary flowering season	April to May	April to May	May to June

In recent years, more than 25 new species of *Sedum* have been reported in China, especially in southwest China. This high level of species richness indicates that this area should be further explored to fully unravel the rich biodiversity there.

### ﻿Taxonomic treatment

#### ﻿ Sedum guangxiense

Taxon classificationPlantaeSaxifragalesCrassulaceae

Yan Liu & C.Y.Zou sp. nov.

B0C8FDB8-8E87-5F50-945E-FDB332FD2DA6

urn:lsid:ipni.org:names:77356943-1

Figs 2 , 3

##### Type.

China • Guangxi: Fangcheng District, Na-suo Town, top of Nanshan. Elevation, 830 m; 21°44'49"N, 108°6'37"E, 24 April 2021. *H. L. Chen ZCY1978* (holotype: IBK!, isotype: PE!).

**Figure 2.**
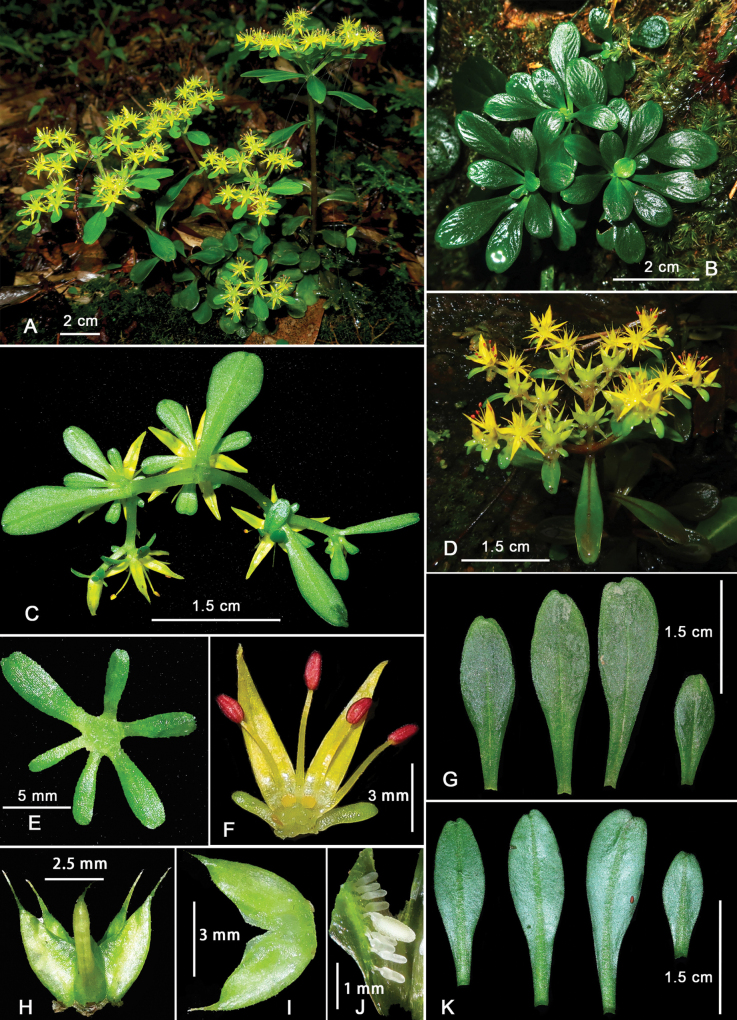
*Sedumguangxiense***A** plant in flower **B** sterile plant **C** inflorescence from below **D** flowering branch **E** strongly unequal sepals **F** side view of a flower with carpels removed, showing the sepals, petals, stamens and nectar scales **G** adaxial leaf surface **H** unripe follicles **I** carpels connate at the base in longitudinal section **J** ovules **K** abaxial leaf surface. A, photographed by Hai-ling Chen in Fangcheng District (corresponding to the holotype *H. L. Chen ZCY1978*); B, C, photographed by Chun-Yu Zou in Yongfu County (corresponding to the paratype *C. Y. Zou & J. Q. Huang, ZCY1977*).

##### Diagnosis.

*Sedumguangxiense* is similar to *S.tosaense* and *S.emarginatum* in its leaf shape and inflorescence type, but can be distinguished from the latter two by its erect stems in youth (vs. prostrate when young), much larger leaves (1.5–3.5 cm long vs. 1–2 cm), narrowly triangular petals (vs. narrowly elliptic-lanceolate and linear-lanceolate to lanceolate), ovate nectar scales (vs. subquadrangular and oblong) and larger, oblong anthers (rather than oblong-ovoid).

**Figure 3.**
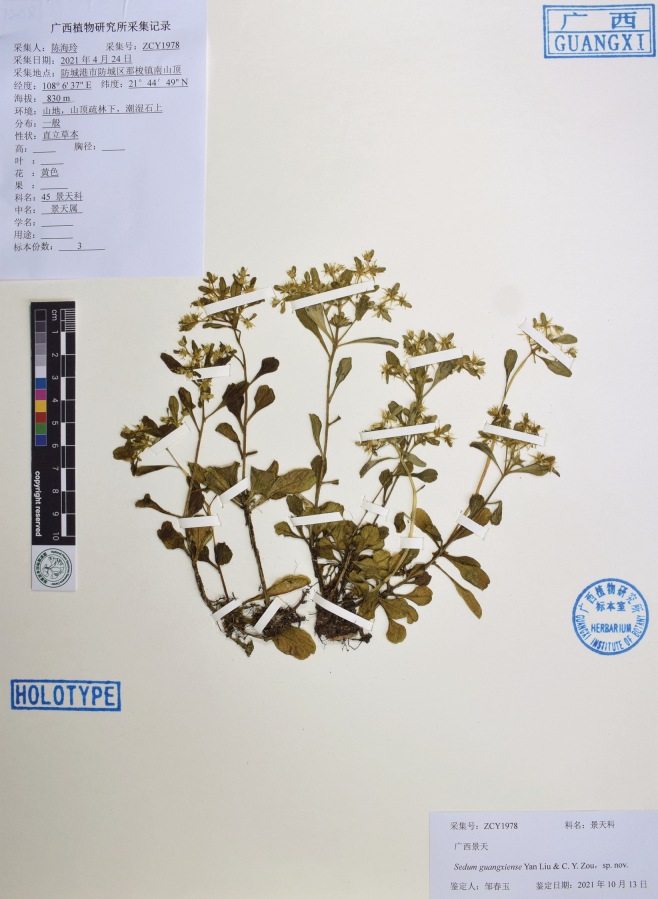
Holotype of *Sedumguangxiense*, *H. L. Chen ZCY1978* (IBK).

##### Description.

Biennial herb, fleshy, glabrous. Roots fibrous, with several adventitious roots in the leaf axils of the basal nodes. Stems stout, erect when young, with irregular branching, to 10–25 cm tall. Leaves alternate, base spurred, sessile, thick, spatulate to obovate, 1.5–3.5 cm long, 0.5–1 cm wide, apex usually retuse, rarely round, base long-attenuate. Inflorescences terminal, many flowered cymes, usually with three branches, each 2-forked; bracts leaf-like, 5–10 mm long, 2–5 mm wide; flowers usually sessile, usually 5-, sometimes 4- or 6-merous; Sepals usually 5, sometimes 4 or 6, free, green, fleshy, strongly unequal in size, spatulate to obovate, 3–8 mm long, 1–3 mm wide, apex usually retuse, rarely round. Petals usually 5, sometimes 4 or 6, yellow, narrowly triangular, 5–6 mm long, 1.1–1.5 mm wide, apex acuminate, slightly connate at base. Stamens usually 10, sometimes 8 or 12, shorter than petals, erect at anthesis, arranged in two whorls; antesepalous ones 3.5–4 mm, antepetalous ones 3–3.5 mm; anthers oblong, ca. 0.8 mm long, reddish-brown before dehiscence. Nectar scales ovate, 0.3–0.4 mm. Carpels usually 5, sometimes 4 or 6, connate at the base for ca. 0.3–0.5 mm, gibbous ventrally, 5–6 mm long; styles 1.6–2 mm long. Fruits star-shaped, many seeded follicles, spreading. Flowering April to May, fruiting May to July.

##### Distribution and habitat.

**Figure 4.**
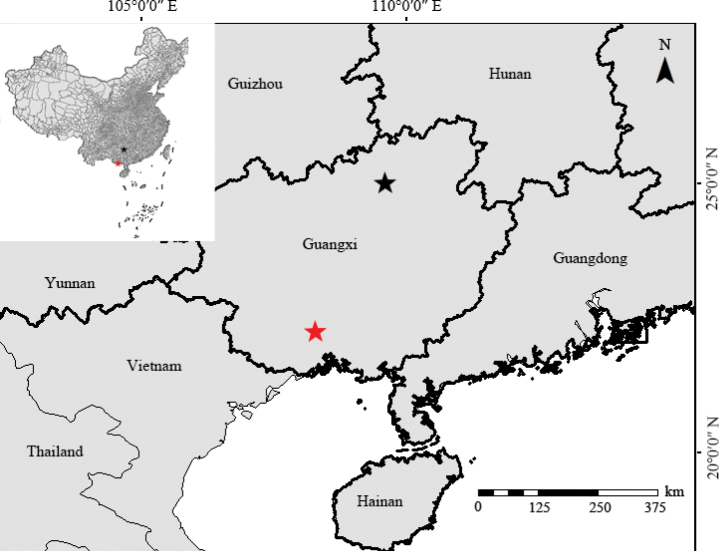
Distribution of *Sedumguangxiense* Yan Liu & C. Y. Zou (pentagram stars) in Guangxi, China. The red pentagram star indicates the locality of the holotype (type locality).

The species is known from Bai-shou Town in Yongfu County and Na-suo Town in Fangcheng District, Guangxi, China. It grows on mossy rocks in secondary broadleaf forests at elevations of 200–550 m (Fig. 4).

##### Etymology.

The specific epithet refers to the distribution in Guangxi Province, China.

##### Additional specimen examined

**(paratypes).** China • Guangxi: Yongfu County, Bai-shou Town, on mossy rocks along streams, elev. 263 m, 25°11'43.29"N, 110°49'42.89"E, 10 May 2021, *C. Y. Zou & J. Q. Huang, ZCY1977* (IBK!); • Fangcheng District, Na-suo Town, the top of Nanshan, 21°44'49"N, 108°6'37"E, 19 April 2021, *Y. G. Liu, Q. G. Yang & H. L. Cheng 1208* (IBK!).

## Supplementary Material

XML Treatment for Sedum guangxiense

## ﻿Additional information

### Author ORCIDs

Yan Liu https://orcid.org/0000-0001-8931-9656

### Data availability

All of the data that support the findings of this study are available in the main text.
